# Insulin resistance and exercise tolerance in heart failure patients: linkage to coronary flow reserve and peripheral vascular function

**DOI:** 10.1186/1475-2840-11-97

**Published:** 2012-08-13

**Authors:** Martin Snoer, Tea Monk-Hansen, Rasmus Huan Olsen, Lene Rørholm Pedersen, Lene Simonsen, Hanne Rasmusen, Flemming Dela, Eva Prescott

**Affiliations:** 1 Dept Cardiology, Bispebjerg University Hospital, Copenhagen, Denmark; 2 Department of Clinical Physiology and Nuclear Medicine, Bispebjerg University Hospital, Copenhagen, Denmark; 3 Xlab Center for Healthy Aging, Department of Biomedical Science, Faculty of Health Sciences, University of Copenhagen, Copenhagen, Denmark

**Keywords:** Coronary flow reserve, Heart failure, Exercise capacity, Insulin sensitivity, Arterial stiffness

## Abstract

**Background:**

Insulin resistance has been linked to exercise intolerance in heart failure patients. The aim of this study was to assess the potential role of coronary flow reserve (CFR), endothelial function and arterial stiffness in explaining this linkage.

**Methods:**

39 patients with LVEF < 35% (median LV ejection fraction (LVEF) 31 (interquartile range (IQ) 26–34), 23/39 of ischemic origin) underwent echocardiography with measurement of CFR. Peak coronary flow velocity (CFV) was measured in the LAD and coronary flow reserve was calculated as the ratio between CFV at rest and during a 2 minutes adenosine infusion. All patients performed a maximal symptom limited exercise test with measurement of peak oxygen uptake (VO_2_peak), digital measurement of endothelial function and arterial stiffness (augmentation index), dual X-ray absorptiometry scan (DEXA) for body composition and insulin sensitivity by a 2 hr hyperinsulinemic (40 mU/min/m^2^) isoglycemic clamp.

**Results:**

Fat free mass adjusted insulin sensitivity was significantly correlated to VO_2_peak (r = 0.43, p = 0.007). Median CFR was 1.77 (IQ 1.26-2.42) and was correlated to insulin sensitivity (r 0.43, p = 0.008). CFR (r = 0.48, p = 0.002), and arterial stiffness (r = −0.35, p = 0.04) were correlated to VO_2_peak whereas endothelial function and LVEF were not (all p > 0.15). In multivariable linear regression adjusting for age, CFR remained independently associated with VO2peak (standardized coefficient (SC) 1.98, p = 0.05) whereas insulin sensitivity (SC 1.75, p = 0.09) and arterial stiffness (SC −1.17, p = 0.29) were no longer associated with VO2peak.

**Conclusions:**

The study confirms that insulin resistance is associated with exercise intolerance in heart failure patients and suggests that this is partly through reduced CFR. This is the first study to our knowledge that shows an association between CFR and exercise capacity in heart failure patients and links the relationship between insulin resistance and exercise capacity to CFR.

## Background

The link between insulin resistance and heart failure is complex. Several studies have shown that insulin resistance is common in heart failure of both ischemic and non-ischemic origin. The exact mechanisms are not known but potentially include both causal and secondary associations. Insulin resistance has been linked to reduced exercise tolerance in heart failure and mechanisms may include both central and peripheral vascular dysfunction [1].

In the absence of significant coronary artery stenosis, coronary flow reserve (CFR) is a measure of microvascular function. CFR has been shown to be reduced in patients with classic risk factors of cardiovascular disease such as hypertension [2], obesity [3] and diabetes [4,5] and is a strong independent marker of poor prognosis in patients with ischemic heart disease [6]. The role of coronary flow reserve in heart failure is less well described. In idiopathic dilated cardiomyopathy CFR has been shown to be reduced [7,8] and in some studies it was also a prognostic marker of future outcome [7,9]. The relationship between insulin resistance and CFR has not been examined in patients with chronic heart failure.

**Figure 1:**
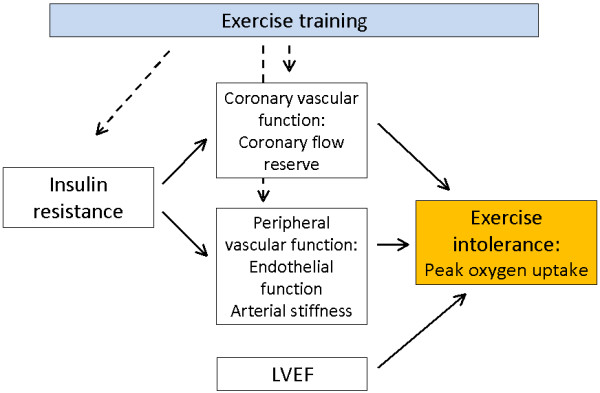
A proposed linkage (non-exhaustive) between insulin resistance, coronary flow reserve, peripheral vascular function, left ventricular ejection fraction and exercise capacity.

In healthy young volunteers CFR has been related to VO_2_peak [10,11]. VO_2_peak is one of the most important prognostic factors in CHF patients [12] and exercise training in heart failure has been shown to improve peak oxygen consumption, muscle strength, symptoms and quality of life and has become part of current guideline recommendations. Effects are thought to be primarily through peripheral vascular and muscular mechanisms [13]. Few studies have examined CFR in relation to VO_2_peak in heart failure patients. Recently, however, in a small randomized trial, 4 months of exercise training of 13 patients with primary heart failure improved both VO_2_peak and CFR [14]. This study added mechanistic insight to explain the apparent benefit of exercise training in heart failure and may also be a link to explain improved prognosis following exercise training in heart failure patients [15]. A possible causal web linking these factors is depicted in Figure 1.

Peak oxygen uptake, vascular function and metabolic function are all important prognostic markers in heart failure. The aim of this study was to assess the potential role of coronary flow reserve, endothelial function and arterial stiffness in explaining the link between insulin resistance and exercise intolerance.

## Methods

### Patients

The study was a local substudy based on patients screened for inclusion to the Smartex-HF trial [16] in which patients with chronic systolic heart failure were randomized to different modalities of exercise training. The patients were recruited from the heart failure outpatient clinic at Bispebjerg University Hospital, Copenhagen, Denmark. Inclusion criteria were a LVEF < 35%, clinically stable (no signs of worsening for at least 6 weeks), minimum 3 months of optimal medical treatment, and if CRT or revascularization was performed, this should be more then 6 months prior to inclusion. All patients had been examined with either coronary angiography or cardiac CT as part of their heart failure examination program and were revascularized according to guidelines. All patients had to have a LAD without significant stenosis (<50%) in order to perform coronary flow measurements. Angiography was not repeated prior to the enrolment in to the study. However, the participants were excluded from the study if there were signs of ischemia or ventricular arrhythmias during the maximal symptom limited bicycle exercise test. The study complied with the Declaration of Helsinki and was approved by the science ethics committee for the Capital Region of Denmark (HC-2008-108). All participants gave informed written consent.

### Cardiopulmonary exercise test

All patients underwent an upright bicycle (Via Sprint 150P, Ergoline, Bitz, Germany) exercise test with breath-by-breath gas exchange measurement (Jaeger, Masterscreen CPX, Cardinal Health, Würzburg, Germany). After 3 minutes of rest on the bicycle the test was initiated using either a protocol starting at 20 watt with a 10 watt increase pr. minute or starting at 40 watts with a 20 watt increase pr. minute, to ensure optimal exercise time based on an expectation of the patients exercise capacity. The patients were encouraged to continue until maximal exhaustion. A leveling off of oxygen uptake despite increasing workload and a respiratory exchange ratio (RER) > 1.05 were used as criteria for maximal oxygen uptake. The mean of the 3 highest consecutive 10-second measurements before exercise terminations were used for determining VO_2_peak.

### Echocardiography

A complete transthoracic echocardiography was performed using Philips IE33 (Philips Medical Systems, Andover, MA, USA) with an S5 probe with the patients in the left supine position. Left ventricle end diastolic (EDV) and end systolic (ESV) volumes and ejection fraction (LVEF) were calculated from apical 2- and 4-chamber views using the biplane Simpson model. Left ventricular mass (LVM) was calculated using the formula LVM = 0.8*(1.04*(LVEDD + PWTd + SWTd)^3^-(LVEDD)^3^) + 0.6 g, where LVEDD is left ventricle end diastolic diameter, PWTd is posterior wall thickness in diastoly and SWTd is septum wall thickness in diastoly, and indexed (LVMi) to body surface area (BSA) calculated by Du Bois’ formula (BSA = 0.007184 x weight kg^0.425^ x height cm^0.725^).

### Coronary flow reserve

**Figure 2:**
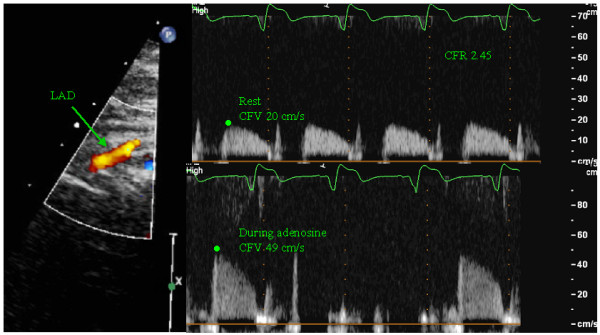
**Coronary flow reserve measurement.** To the left a color Doppler image of the mid-distal part of the LAD. The top image shows the PW recording of the blood flow velocity in the LAD during rest and the bottom shows blood flow during adenosine infusion. The scale has been changed from rest to hyperemia. The marking represents peak diastolic flow velocity. Here the CFR is 2.45.

CFR can be measured non-invasively using transthoracic Doppler echocardiography with a high success rate [17] and this technique has been validated against invasive measurements [18] with good results. CFR was measured using a high frequency broadband transducer (S8, Philips). All patients were instructed to abstain from caffeine for 12 hours before the examination and oral use of dipyridamole was paused for 72 hours. With the patient in the left supine position the LAD was located as distal as possible using color Doppler either in an apical modified two chamber view or middistal using a modified short axis view. Coronary flow velocity (CFV) was measured using pulsed wave Doppler with a sample size of 3–4 mm, at rest and during a 2 minute infusion of adenosine at 0.14 mg kg^-1^ min^-1^. The solution was diluted so that the infusion rate was kept at 10 ml min^-1^. Before and during the adenosine infusion care was taken to maintain the position and angle of the probe, so measurements were done on the same segment of the LAD at the same angle. During the infusion the scale of flow velocity was changed in order to obtain optimal curves for offline measurement. CFR was calculated as the ratio between peak diastolic CFV during adenosine infusion and during rest using a mean of 3 consecutive cardiac cycles (10 cycles if the patient had atrial fibrillation). Analyses were done offline by an investigator blinded to the other examinations. Blood pressure and ECG were monitored before and during the adenosine infusion. Interobserver variability of CFR was tested on all subjects and the mean difference in CFR was 0.07 with limits of agreement ±0.21. The coefficient of variation (CV), calculated as the within-subject standard deviation divided by the mean of the observations, was 5.5%. Intraobserver variability was tested on 10 randomly selected examinations with a mean difference of 0.03, limits of agreement ±0.29 and CV 7.5%. Figure 2 shows measurement of CFR

### Insulin sensitivity

Insulin sensitivity was measured using an isoglycemic hyperinsulemic glucose clamp, with an insulin infusion rate at 40 mU/min/m^2^. Subjects met in the morning after a 12-hour overnight fast and without unusual strenuous activity for 3 days before the clamp. Blood glucose concentrations were measured in arterialized blood drawn from a catheter inserted in a dorsal hand vein. Arterialisation of the blood was achieved by placing the hand in a heating pad. Fasting blood glucose concentration was determined as the average of two blood samples, taken at t = −15 min and 0 min. Insulin was initiated by a bolus injection, and then infused continuously for 120 minutes. Glucose was measured every 5 minutes using a handheld glucose-measuring device (Accu-chek Inform, Roche Diagnostics, Indianapolis, USA). Insulin sensitivity was defined as the steady-state average glucose infusion rate during the final 30 minutes of the clamp (M-value). Metabolic clearance rate of glucose was calculated as the M-value divided by the prevailing blood-glucose concentration in order to facilitate comparisons between individuals at the steady-state condition.

### Body composition

A whole body dual x-ray absorptiometry scan (DEXA) (Lunar DPX-IQ, GE Lunar Corp, Madison, WI) was performed for estimation of body composition (fat mass and fat free mass).

### Vascular function

Flow mediated vasodilation measurement was performed using Endo-PAT 2000 (Itamar, Israel) which measures arterial pulsatile volume changes in the fingertip before and after upper arm occlusion. The examination was performed in the morning in a fasting state. After 5 minutes of baseline measurements upper arm occlusion on one arm was sustained for 5 minutes using a blood pressure cuff inflated to a minimum of 200 mmHg, while the other arm served as a control. After the release of the cuff the hyperemic response was recorded and the reactive hyperemia index (RHI) was calculated as a measure of endothelial function using the automatic software taking the relative difference between basic and hyperemic blood flow on the occluded arm and dividing it with the response on the control arm, to correct for any autonomic changes in vascular diameter.

Augmentation index is derived from pulse wave analyses and is a measure of arterial stiffness. A higher augmentation index indicates more arterial stiffness [19]. Data was collected using the Endo-PAT 2000, where pulse waveforms from the digital probes obtained before upper arm occlusion were used to calculate the heart rate so the results are normalized for heart rate at 75 bpm and were calculated using the Endo-PAT 2000 software.

### Statistics

Unless stated otherwise values are expressed as median and inter quartile range for continuous variables and as number (percent) for categorical variables. Continuous variables were compared using Students unpaired *t*-test and differences in categorical variables were assessed using chi-square test. Linear regression was used to test the relationship between variables and correlations were calculated using Pearsons correlation coefficient. A multivariable linear regression using standardized coefficients (SC) was done to identify independent predictors of VO_2_peak. A P value of less than 0.05 was considered statistically significant. All analyses were performed in STATA 11.1 (StataCorp. 2009. Stata Statistical Software: Release 11. College Station, Texas, USA).

## Results

Patient characteristics are displayed in Table 1. A total of 39 patients (33 men), without significant stenosis of LAD were included and had a successful measurement of CFR. Median LVEF was 31 (IQ range 26–34) and 23/39 were of ischemic origin.

**Table 1:** Baseline patient characteristics. Values are median (IQ range) or number (%) as indicated

**Patient characteristics**	**Median (IQ range)**	
Age	65	(58–76)
Male sex	33	(84.6%)
BMI	26.7	(23.8-29.6)
Fat %	28.9	(20.7-33.7)
ICD	13	(33%)
Diabetes	8	(20.5%)
Atrial fibrillation	7	(18%)
Mitral regurgitation	22	(56%)
Ischemic ethiology	23	(59%)
Previous MI	19	(49%)
- involving the LAD	17	(44%)
Previous PCI	15	(38%)
- involving the LAD	13	(33%)
Previous CABG		
- all involving the LAD	11	(28%)
LVEF %	31	(26–34)
NYHA		
▪→II	32	(82%)
▪→III	7	(18%)
Medication		
▪→ACE-inhibitor or ARB	36	(92%)
▪→Beta-blockers	37	(95%)
▪→Loop-diuretics	22	(56%)
▪→Spironolacton	18	(46%)

BMI: Body mass index, ICD: Implantable cardioverter-defibrillator, MI: myocardial infarction, LAD: left anterior descending artery, PCI: percutaneous coronary intervention, CABG: coronary artery bypass graft, LVEF: left ventricular ejection fraction, NYHA: New York Heart Association, ACE: Angiotensin converting enzyme, ARB: Angiotensin-2-recepter blockers.

### Exercise capacity

Median VO_2_peak was 16.2 (14.6-19.4) using total body mass and 23.5 (20.1-27.4), when using fat free mass in the calculation. No ventricular arrhythmias or signs of ischemia were seen during the test. Median test duration was 7.2 minutes (6.0-8.7) and maximum watt achieved was 90 watts (70–120). Median RER was 1.14 (1.07-1.19). VO_2_peak (FFM adjusted) was higher in the patients with non-ischemic vs. ischemic heart failure (median 26.2 vs. 22.5, p = 0.05). There were no differences between patients with or without diabetes and atrial fibrillation.

### Insulin sensitivity

Median M-value was 4.8 mg/min/kg (3.6-5.6) and glucose clearance was 4.2 ml/min/kg (3.3-5.1) when using total body mass and 6.8 mg/min/kg FFM (4.9-8.1) and 6.0 ml/min/kg FFM (4.7-7.2) respectively when using fat free mass. When comparing patients with insulin sensitivity above and below the median value 6.8 (Table 2) the patients with higher insulin sensitivity had significantly higher CFR and tended to have higher VO_2_peak (p = 0.09), but otherwise there were no differences.

**Table 2:** Measurements for patients divided into groups with low and high CFR and insulin sensitivity by the median

	**Coronary flow reserve**				**Insulin sensitivity**			
	**Low**		**High**		**Low**		**High**	
	**CFR < 1.77**		**CFR > 1.77**		**Insulin sensitivity**		**Insulin sensitivity**	
	**n = 19**		**n = 20**		**n = 19**		**n = 20**	
**Exercise capacity**								
VO_2_peak (ml/min/kg FFM)	22.1	(19.1-25.5)	26.3	(21.1-29.7)^*^	22.1	(19.9-26.4)	25.5	(20.3-29.5)
RER	1.14	(1.07-1.19)	1.11	(1.03-1.19)	1.12	(1.07-1.19)	1.13	(1.06-1.16)
**Vascular function**								
RHI	1.51	(1.34-1.91)	1.59	(1.37-1.92)	1.54	(1.40-1.67)	1.62	(1.33-2.36)
Augmentation Index	12.4	(−1.6-32.8)	11.0	(−5.7-16.6)	12.8	(−3.4-27.6)	8.1	(−6.3-21.2)
**Glucose metabolism**								
Insulin sensitivity (mg/min/kg FFM)	5.8	(4.4-6.9)	7.2	(6.4-9.2)^*^	4.9	(3.5-6.3)	8.1	(7.1-9.2)^§^
Glucose clearence (ml/min/kg FFM)	5.1	(4.4-5.9)	6.6	(5.6-8.9)	4.7	(2.8-5.8)	7.2	(6.3-9.3)^§^
**Microvascular function**								
CFV rest (cm/s)	29.7	(23.0-33.0)	22.7	(18.5-27.3)^§^	30.0	(22.0-33.0)	23.0	(19.0-25.3)^*^
CFV stress (cm/s)	35.0	(29.0-43.0)	63.3	(43.0-72.2)^§^	41.3	(32.3-63.6)	47.3	(37.0-65.7)
CFR	1.26	(1.04-1.55)	2.39	(2.09-3.09)^§^	1.41	(1.04-2.14)	2.10	(1.70-3.09)^§^
**Echocardiography**								
LVEF (%)	29.5	(23–34)	31.5	(27–33)	31	(26–33)	31	(26–34)
EDV (ml)	183	(144–245)	153	(112–219)	183	(113–255)	172	(138–219)
ESV (ml)	132	(95–176)	104	(75–159)	127	(77–176)	122	(90–155)
LVMi (g/m^2^)	131	(110–148)	104	(82–159)	128	(92–148)	126	(89–152)

CFR: coronary flow reserve, VO_2_peak: peak oxygen uptake, RER: Respiratory exchange ratio, RHI: Reactive hyperaemia index, FFM: Fat free mass, LVEF: left ventricular ejection fraction, EDV: left ventricle end diastolic volume, ESV: left ventricle end systolic volume, LVMi: left ventricle mass index * p < 0.05, § p < 0.01

Values are expressed as median (IQ range).

### Coronary flow reserve

Median CFR was 1.77 (1.26-2.42), and there were no significant differences in CFR between patients of ischemic and non-ischemic origin (1.57 vs. 1.99, p = 0.48), patients with or without type 2 diabetes (1.59 vs. 1.88, p = 0.31), with or without mitral regurgitation (1.67 vs. 1.88, p = 0.24) or between NYHA group II and III (1.78 vs. 1.42, p = 0.48). There were also no differences in patients with or without previous myocardial infarction, percutaneous coronary intervention (PCI) and coronary artery bypass graft (CABG) operation regardless of whether the LAD was involved or not (all p > 0.05). When comparing high and low CFR divided by the median (Table 2), patients with higher CFR had higher VO_2_peak and insulin sensitivity, but did not differ with regards to peripheral vascular factors. The patients with higher CFR tended to have lower EDV, ESV and LVMi, but this was not statistically significant.

**Figure 3:**
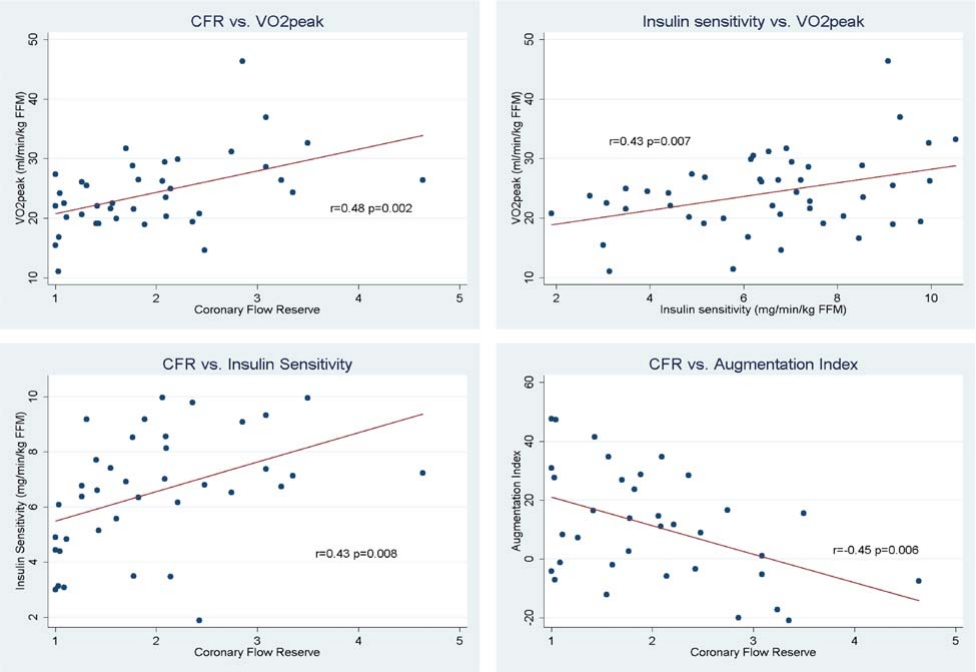
**Scatter plots with linear regression lines between VO**_**2**_**peak and CFR (upper left), VO**_**2**_**peak and insulin sensitivity (upper right), insulin sensitivity and CFR (lower left) and augmentation index and FR (lower right).**

Table 3 shows correlations between the different measurements. VO_2_peak was correlated to insulin sensitivity (r = 0.43, p = 0.007), CFR (r = 0.48, p = 0.002), augmentation index (r = −0.35, p = 0.04) and age (r = −0.38, p = 0.02) (Figure 3). There was a correlation between CFR and insulin sensitivity (r = 0.43, p = 0.008) and augmentation index (−0.45, p = 0.007). The relationship between insulin sensitivity, CFR and VO_2_peak was still present when removing the 8 patients with type 2 diabetes (r = 0.48, p = 0.006 and r = 0.49, p = 0.006 respectively). There were no significant correlations between resting (r = −0.29, p = 0.07) and hyperemic (r = 0.29, p = 0.07) CFV and VO_2_peak. Insulin sensitivity was correlated to resting (r = −0.33, p = 0.04) but not to hyperemic (r = 0.20, p = 0.22) CFV. There was no significant correlation between CFR and EDV, ESV and LVMi.

**Table 3:** Correlations and p-values between different measurements

	**Age**		**Augmentation index**		**RHI**		**Insulin sensitivity**		**LVEF**		**CFR**	
VO2peak	−0.37	(0.02)	−0.35	(0.04)	−0.24	(0.16)	0.43	(0.007)	0.23	(0.15)	0.48	(0.002)
CFR	−0.18	(0.27)	−0.45	(0.007)	−0.10	(0.56)	0.43	(0.008)	0.18	(0.26)		
LVEF	0.05	(0.76)	0.12	(0.48)	0.08	(0.65)	0.14	(0.41)				
Insulin sensitivity	−0.14	(0.41)	0.12	(0.50)	0.13	(0.45)						
RHI	−0.03	(0.85)	0.44	(0.008)								
Augmentation index	0.14	(0.44)										

RHI: Reactive hyperemia index, LVEF: Left ventricular ejection fraction, CFR: Coronary flow reserve.

Unlike augmentation index endothelial function measured by Endo-Pat 2000 was not correlated with CFR (r = −0.10, p = 0.56), insulin sensitivity (r = 0.13, p = 0.45) or VO_2_peak (−0.24, p = 0.16). Left ventricular ejection fraction was not correlated to CFR (r = 0.18, p = 0.26), VO_2_peak (r = 0.23, p = 0.15) or insulin sensitivity (r = 0.14, p = 0.41).

In multivariable linear regression adjusting for age (Table 4), CFR remained independently associated with VO_2_peak (SC 1.98, p = 0.05) whereas insulin sensitivity (SC 1.75, p = 0.09) and arterial stiffness (SC −1.17, p = 0.29) were no longer associated with VO_2_peak. The same results were achieved when using glucose clearance instead of insulin sensitivity.

**Table 4:** **Univariate and multivariable linear regression with VO**_**2**_**peak as the dependent factor**

	**Univariate beta**	**p**	**Multiple beta**	**p**
Age	−0.23	0.02	−0.18	0.04
Insulin sensitivity (SC)	2.86	0.007	1.75	0.09
CFR (SC)	3.07	0.002	1.98	0.05
RHI (SC)	−2.01	0.16		Ns
Augmentation index (SC)	−2.21	0.04		Ns
LVEF	0.25	0.15		Ns

SC: Standardized coefficient, CFR Coronary flow reserve, RHI: Reactive hyperemia index, LVEF: Left ventricular ejection fraction.

## Discussion

The present study confirms that exercise intolerance is related to insulin resistance in heart failure patients. The study further shows that coronary flow reserve, a measure of coronary microvessel function, is correlated to both exercise capacity and insulin resistance and supports a mediating role of CFR in explaining the link between insulin resistance and exercise intolerance. Peripheral endothelial function and arterial stiffness were not associated with exercise capacity and, notably, left ventricular function was not related to either insulin resistance, exercise capacity, coronary- or peripheral vascular function.

Previous studies has shown a reduced CFR in patients with type 2 diabetes compared to patients without diabetes and an association between CFR and the severity of diabetes measured as HbA1c [3] and fasting glucose [5]. Only two studies have examined the relationship between the degree of insulin resistance and CFR in patients without diabetes, with one study showing an inverse relationship between CFR and HOMA index in 45 women with suspected coronary artery disease and angiographically normal coronary arteries [20] and another showing correlation between CFR and insulin sensitivity measured using a hyperinsulinaemic euglycemic clamp in obese patients [21]. Patients with chronic heart failure are known to have a high degree of insulin resistance and many develop diabetes. The mechanisms of this are unclear, but a common denominator in type 2 diabetes and heart failure is physical inactivity. In the present study patients with type 2 diabetes did not have significantly reduced CFR, although with a larger study population there might have been a difference, but insulin sensitivity was strongly correlated with CFR, indicating pre-diabetes microvascular damage.

Exercise intolerance is the most prevailing symptom in chronic systolic heart failure and VO_2_peak is an important prognostic factor in this patient group. However, the link between the degree of cardiac dysfunction and exercise capacity is poorly understood, and previous studies have shown a poor correlation between VO_2_peak and LVEF [22]. In concordance, we found no correlation between these two parameters. We found a positive correlation between CFR and VO_2_peak, which has been shown previously in healthy young men but not in patients with heart failure [10,11]. It is possible that impaired microvascular function contributes to explain the link between cardiac dysfunction and the impairment inVO_2_peak.

Insulin resistance is highly prevalent in heart failure [23]. In accordance with previous studies we found a positive correlation between insulin sensitivity and VO_2_peak in heart failure patients [1,24], which remained unaffected when leaving out patients with diabetes. The underlying mechanism for the relationship between insulin sensitivity and VO_2_peak is not completely understood. We showed that when adjusting for the effect of CFR on VO_2_peak, the relationship to insulin sensitivity was weakened and no longer statistically significant. A possible explanation is that increasing insulin resistance influences the cardiac microcirculation resulting in a reduced CFR, which leads to an impaired exercise capacity (Figure 1).

Our study did not show any relationship between CFR and peripheral endothelial function measured using flow mediated vasodilation. While a relationship has been shown between CFR and flow mediated vasodilation using brachial ultrasound in other patient populations [25], this is not well examined in heart failure patients. In one study of patients with dilated cardiomyopathy no correlation between CFR using echocardiography and flow mediated vasodilation was found [26]. Similarly, a study comparing patients with non-ischemic heart failure to healthy controls found that flow mediated vasodilation and CFR measured using positron emission tomography were correlated in the healthy controls but not in the patients with heart failure [27]. Interestingly, in a small study of dogs with pacing induced heart failure, CFR was reduced with progression of heart failure, but coronary endothelial function was preserved until a late stage of heart failure [28]. Thus our results are consistent with the literature in finding no association between CFR and endothelial function.

The present study shows a negative correlation between CFR and augmentation index, which is as measure of stiffness in the large arteries. Only one previous study has related CFR to augmentation index [29]. In this study of asymptomatic patients with risk factors for ischemic heart disease, the patients with higher augmentation index and pulse wave velocity had a lower CFR. In a normal elastic aorta the pulse wave reflects from the periphery and returns to the heart in diastole, which improves the diastolic filling of the coronary arteries. With aorta stiffening the pulse wave returns during systole resulting in increased afterload and myocardial oxygen demand which could mean, that a reduced CFR in heart failure patients without significant coronary artery stenosis might not only be due to impaired coronary microcirculation, but that a reduced diastolic filling can be a participating factor. Insulin sensitivity and augmentation index were independently associated with CFR, suggesting that they influence CFR through different mechanisms. However, this finding remains to be confirmed in other studies.

Previous studies have shown that about 50% of asymptomatic patients with type 2 diabetes have signs of diastolic dysfunction [30] and that diastolic dysfunction is associated with insulin resistance in patients with suspected coronary artery disease without diabetes [31] without systolic heart failure. In patients with systolic heart failure, patients with type 2 diabetes have significantly higher E/e’ than patients without diabetes [32]. Although we did not include measures of diastolic function the present study, a possible mechanism between insulin sensitivity and diastolic dysfunction might be impaired coronary microvascular function.

A reduced CFR has been shown to be an independent predictor of poor outcome in patients with dilated cardiomyopathy [8] and in a mixed population of heart failure patients [33]. In the present study we find a relationship between CFR, VO_2_peak and insulin sensitivity, which also are well known prognostic risk factors in heart failure, suggesting that these risk factors are closely linked. Improving VO_2_peak and insulin sensitivity through exercise training could also improve CFR as has been shown in a recent study of heart failure patients [14]. CFR has also been improved through other interventions such as weight loss in obese women [34] and after treatment with a beta-1 receptor blocker in dilated cardiomyopathy [35]. However, it has not yet been shown whether improvement in CFR through e.g. exercise training is followed by an improvement in prognosis for heart failure patients. An ongoing study will determine if CFR can improve after different modalities of exercise training in heart failure patients, and if any changes will correspond to changes in peak oxygen uptake, metabolic fitness and in augmentation index.

Limitations: Although the study is limited in size this is somewhat counteracted by the precision of the measures used: insulin sensitivity and glucose clearance were assessed by hyperinsulinemic isoglycemic clamp, the gold standard, and adjusted to fat free mass from DEXA scan. VO_2_peak was a true maximum test as indicated by the high RER values. The cross-sectional nature of the study impedes causal inference and any conclusions regarding potential effects of intervention must therefore remain speculative. The patients had all had a LAD without significant stenosis at a previous coronary angiography or coronary CT-scan, but the examination was not repeated at the time of the study, meaning that some of the patients potentially could have developed a significant LAD-stenosis. All of the patients did however perform a maximal symptom limited cardiopulmonary exercise test at enrolment in to the study without chest pain or signs of ischemia on the ECG minimizing the chances of a significant LAD-stenosis.

## Conclusion

Our study confirms that insulin resistance is associated with reduced exercise tolerance in heart failure patients and suggests that this is partly due to reduced coronary flow reserve. This is the first study to our knowledge that shows an association between CFR and exercise capacity in heart failure patients and links the relationship between insulin resistance and exercise capacity to CFR.

## Abbreviations

AI, Augmentation index; BSA, Body surface area; CFR, Coronary flow reserve; CFV, Coronary flow velocity; DEXA, Dual X-ray absorptiometry; EDV, End diastolic volume; ESV, End systolic volume; FFM, Fat free mass; ICD, Implantable cardioverter-defibrillator; IQ, Inter quartile; LAD, Left anterior descending artery; LVEF, Left ventricular ejection fraction; LVMi, Left Ventricular Mass index; NYHA, New York Heart Association; RER, Respiratory exchange ratio; RHI, Reactive hyperemia index; SC, Standardized coefficient; VO2peak, Peak Oxygen uptake.

## Competing interests

The authors declare that they have no competing interests.

## Authors’ contribution

MS participated in the design of the study, collection of data, statistical analysis and drafting the paper. TM participated in the design of the study, collection of data and contributed to discussion. RO participated in analyzing the data and contributed to discussion. LP participated in analyzing the data and contributed to discussion. LS reviewed/edited the manuscript and contributed to discussion. HR reviewed/edited the manuscript and contributed to discussion. FD participated in the design of the study, reviewed/edited the manuscript and contributed to discussion. EP participated in the design of the study, statistical analysis reviewed/edited the manuscript and contributed to discussion. All authors read and approved the final manuscript.

## Funding

This work was supported by The Danish Research Council.
